# Health Insurance Notes

**Published:** 1923-09

**Authors:** 


### NATIONAL HEALTH INSURANCE NOTES.

### THE CAPITATION FEE.

The question of the amount of the payment to be made to the doctors after December 31st next overshadows at the moment every other question in the Health Insurance field. It is dealt with elsewhere in a special article. Since that article was written the Insurance Acts Committee of the British Medical Association have presented to the Minister, on behalf of the profession, a Memorandum containing a considered statement of the doctors' case for a capitation fee of from 10s. 4d. to 10s. 9d. This is placed as the lowest figure for "securing a really adequate general practitioner service of a high type." There will inevitably be a disposition to read this sentence with a stress on the word "really," and to reflect that, while there is every desire that the really good doctor should be well paid, the capitation fee must be paid alike to good, bad, and indifferent doctors. Perhaps the most arresting feature about the document is the statement based on a Memorandum prepared by Professor Bowley that the change in the cost of living since January, 1920, would justify a drop of 4 per cent. only from the 11s. fee fixed after arbitration at that date.

### THE COUNTRY DOCTOR.

The really hard case of the country doctor with a poor, sparsely populated district can be fully met at such a relatively insignificant cost, compared with the total sum paid to doctors, that there will assuredly be a generous reception of that part of the doctors' case representing that "some additional financial assistance is required for practitioners working in certain rural areas." This is a statement from a summary of the Insurance Acts Committee's proposals. It refers, it will be seen, to "certain" rural areas, and to that extent would appear to emphasise the suggestion in the body of the document that "there should be established a relatively small fund analogous to the Lowlands Necessitous Districts' Grant (in Scotland) to give assistance in certain respects to practitioners in single-practice sparsely populated areas, the needs of each case being individually investigated." But there is also in the document a suggestion that the general mileage payment should be revised so as to take account of travelling in rural areas within two miles of the doctor's house; this is treated, at present, as corresponding more or less to a similar radius of travelling in an urban area. There are practical difficulties, mainly of definition, in this matter, but it may be assumed that the Minister will give this part of the case also a most sympathetic consideration.

### THE RANGE OF SERVICE.

The doctors have very wisely included in their representations the following:—"That this opportunity should be taken of establishing further diagnostic facilities (laboratory and X-ray) for the benefit of insured persons, and that consideration should at once be given to the question of the provision and administration of other extensions of the medical service." From the national standpoint, it is unquestionable that an organised scheme of extensions of the Medical Service should be made the subject of a charge on the National Health Insurance Fund as a whole, rather than that surpluses should accumulate to be disposed of in various ways at the whim or fancy of the members or the executives of particular societies. We understand that there is a disposition on the part of Societies to avoid in the future the frittering away of surpluses on cash payments. This is all to the good, but where a scheme is so largely financed by employers and by the Exchequer, and all contributions are compulsory, there should be national, rather than sectional, organisation of the scheme of benefit. It will be a great opportunity missed if the Minister is not able to make some hopeful preliminary announcement on this question when meeting the doctors.

### THE SURPLUSES OF SOCIETIES.

Considerable support for the statement that there is a disposition on the part of societies to avoid in future wasting their surpluses in cash payments to their members will be found on a perusal of the report of the discussion at the Foresters' High Court just held at Portsmouth. Several speakers were "whole-hoggers" in the matter of giving "treatment" rather than cash benefits in future, and they supported the view that the attention of the Minister should be drawn to the urgent necessity for requiring every approved society and branch to utilise surpluses in providing benefits in kind, "instead of permitting such surpluses to be utilised solely in augmenting the statutory cash benefits." It is not difficult to understand why the Foresters should want the Minister to take general action in the matter. There is competition for new members among the societies, and if it is known that one society can give very definite and tangible evidence of its profits in the form of additional cash payments, it is doubtless able to attract more members than if it utilises its profits in providing extra treatment in some form. This is not at all as it should be, and we are glad to see thoughtful and earnest members making a stand for the consideration of the best means of disposing of surpluses by reference solely to the well-being of the whole body of members.

### THE TREATMENT OF SEAMEN.

In the columns of the *British Medical Journal*, recently, panel doctors have been informed that the Seamen's National Health Insurance Society arranges direct with individual doctors, and not with the Insurance Committees, to give medical treatment to their members, and that, accordingly, a panel doctor not under such agreement with the Society is under no obligation to treat an applicant who is a member of the Society. It is true, therefore, as the *Journal* points out, that the panel doctor may charge such an applicant a fee as a private patient. We think, however, that a doctor would be well advised not to do so, but to send his account to the Society at Leman Street, London, E., and accept payment at their usual rates.

